# Randomized, Double-Blind, Placebo-Controlled Study of Modified Erzhi Granules in the Treatment of Menopause-Related Vulvovaginal Atrophy

**DOI:** 10.1155/2018/6452709

**Published:** 2018-10-14

**Authors:** Ranran Chen, Dianrong Song, Wei Zhang, Guanwei Fan, Yingqiang Zhao, Xiumei Gao

**Affiliations:** ^1^Tianjin University of Traditional Chinese Medicine, China; ^2^The Second Affiliated Hospital of Tianjin University of Traditional Chinese Medicine, China

## Abstract

**Objective:**

To evaluate the clinical therapeutic efficacy and safety of modified Erzhi granules (MEG) in patients with menopause-related vulvovaginal atrophy (VVA).

**Methods:**

This randomized, double-blind, placebo-controlled study comprised two groups, including the treatment and control groups. Patients receive MEG and placebo for 12 weeks, respectively. Vaginal health score (VHS), vaginitis score, vaginal maturation index (VMI), female sexual function index (FSFI), and modified Kupperman Index (modified KI) were used as efficacy endpoints and assessed at baseline, 4, 8, and 12 weeks during administration, and 4 weeks after drug withdrawal. At baseline and 12 weeks, serum estradiol (E_2_), follicle stimulating hormone (FSH), pelvic ultrasound, breast ultrasound, and other safety parameters were measured, recording adverse events.

**Results:**

At 12 weeks, VHS, percentage of superficial cells in the vaginal epithelium and FSFI were significantly increased, while vaginitis score, percentage of basal cells in the vaginal epithelium, and modified KI were significantly decreased in comparison with baseline and control group (all P<0.05); these differences persisted for up to 4 weeks after drug withdrawal. The placebo group showed no significant change during treatment compared with baseline values (*p*>0.05). Serum E_2_ and FSH levels, endometrial thickness, and breast thickness in all patients were within the normal ranges before and after treatment, with no serious adverse reactions observed.

**Conclusion:**

MEG significantly alleviates menopause-related vulvovaginal atrophy, with no overt adverse effects on the endometrium, breast, hepatic, and renal functions.

## 1. Introduction

Vulvovaginal atrophy (VVA), which often occurs 2-5 years after menopause [1], is a common disease in postmenopausal women, with an incidence of up to 50% [2]. VVA is mainly due to decreased estrogen levels after menopause, which reduces the thickness of epithelial tissues in the female reproductive tract, smooth muscle function, and collagen and hyaluronic acid levels; this results in decreased vaginal wall elasticity, increased mucosal fragility, imbalanced local flora, and increased pH. The clinical manifestations include vaginal dryness, painful sexual intercourse, vaginal burning, postcoital bleeding [3]. Vaginal wall atrophy, flattened villi, loss of folds, and pale pink petechiae are occasionally found in gynecologic examination. Because pelvic floor muscles, the urethra, and the urinary bladder's trigone are also rich in estrogen receptors, connective tissue relaxation and sphincter dysfunction occur with declined body hormone levels, often accompanied by frequent urination, urinary urgency, urinary pain, enuresis, recurrent urinary tract infection, and other symptoms. According to a survey, 44.4% of women with VVA have moderate or severe vaginal dryness, with 30.2% of women with VVA experiencing severe pain during intercourse [4].

Another study in the United States found that VVA not only causes local discomfort in the vagina, but also significantly affects the patient's sexual function, sleep pattern, and personality [5]. A survey of women whose ages are between 55 and 65 revealed that 58% of patients with vaginal discomfort avoid intimacy [6]. Moreover, vaginal itching, leukorrhea, and sexual problems caused by VVA seriously affect the quality of life of postmenopausal women [7].

Hormone replacement is currently considered the most effective treatment [8]. Meanwhile, lubricants, vaginal flora regulators, and androgen preparations are also widely used in clinical practice. However, the safety of hormone replacement therapy remains controversial, especially for patients with a history of breast or endometrial cancer, or those with atherosclerotic heart disease, venous thromboembolism, or active liver disease. In addition, no consensus has been reached regarding the optimal treatment scheme and dose in hormone replacement therapy [9]. Vaginal lubricant treatment increases the rate of sexually transmitted diseases (*p*=0.006) and adversely affects vaginal epithelial cells and flora [10, 11]. Moreover, it also tends to cause mucosal irritation (*p*=0.001), resulting in further aggravation of long-term symptoms [12]. The efficacy and safety of other drugs in the treatment of VVA remain largely undefined.

Traditional Chinese medicine suggests that the relationship between VVA occurrence and the kidney is the closest; the syndrome of Yin deficiency of kidney is mostly in the perimenopausal and early menopausal periods; with the prolongation of menopausal time, the innate essence is further exhausted, Yang cannot support Yin, and Yin impairment affects Yang, leading to kidney Yang debilitation. The main clinical manifestations are cold, declined sexual desire, soreness and weakness of waist and knees, vaginal dryness, and dysuria.

Our previous studies [13, 14] found that, compared with the placebo group, treatment with the formulation comprising Jiawei Qing'e Fang, Danzhi Qing'e formula, and Erzhi formula effectively improves the quality of life of perimenopausal women and relieves vasomotor symptoms. Therefore, we aimed to further assess the clinical efficacy of this traditional Chinese medicine on menopause-related VVA.

The MEG is a compound preparation based on our previous clinical and pharmacological studies and includes* Fructus Ligustri Lucidi*,* Eclipta*,* Herba Cistanches, Cynomorium Songaricum,* and* Cortex Phellodendri*. A randomized, double-blind, placebo parallel control trial was conducted to assess the efficacy and safety of MEG on menopause-related VVA by observing changes in patient's VHS, vaginitis score, VMI, FSFI, and modified KI.

## 2. Materials and Methods

### 2.1. Ethics and Registration

This study was approved by the ethics committee of the Second Affiliated Hospital of Tianjin University of Traditional Chinese Medicine and registered in the Chinese Clinical Trial Registry (Registration Number: ChiCTR-IOR-16009312). All the patients included provided signed informed consent before enrolment.

### 2.2. Principle of Study Design

#### 2.2.1. Sample Content

Sample size for superiority trials for comparing two means was used to evaluate the formula for calculations.

It was expected that, after 12 weeks of treatment, the difference in VHS between test group and control group would be 4 points, for a total variance of 2.75. The optimal threshold value for statistical superiority was set at Δ=3. Class I and II errors were set at *α*=0.025 and *β*=0.10, respectively; therefore, at least 35 cases were required in each group.

#### 2.2.2. Randomization

A completely randomized design was adopted; the random arrangement for treatments (therapeutic and control drugs) of subjects 01 to 88 was obtained with the SPSS software, which generated a random coding table.

#### 2.2.3. Blinding Method

This was two level of blind design. The first level was processing a code corresponding to the groups (group A or B); the second level was the drug corresponding to the processing code. All the drug encoding process was written in the form of a document (blind code record) by a code-blinded person, which was kept as one of the clinical trial documents. The contents included preparation of drugs, drug packaging, prescription, storage requirements, drug delivery method, generation of random processing, drug packaging box for each subject, emergency letter, preservation of treatment code, and regulation of unblinding. The two levels of treatment codes were sealed separately and conserved by the Experimental Center and Research Department of the Second Affiliated Hospital of Tianjin University of Traditional Chinese Medicine. There was an emergency letter corresponding to each coded trial drug for unblinding in case of emergency.

### 2.3. Subjects

The subjects were eligible patients treated from November 2016 to April 2017 at the gynecologic outpatient clinic of the Second Affiliated Hospital of Tianjin University of Traditional Chinese Medicine.

Inclusion criteria were ① age from 45 to 65 years, with menopause time ≥1 year; ② at least one of VVA symptoms, such as vaginal dryness, vaginal or genital itching, painful sexual intercourse and so on; ③ percentage of superficial cells in the vaginal epithelium ≤10% and vaginal pH>5; ④ follicle stimulating hormone (FSH) ≥40 mIU/ml and estradiol (E_2_) ≤20 pg/ml; ⑤ signed informed content. Exclusion criteria were: ① a history of malignant tumor of the reproductive system or breast cancer; ② unexplained uterine bleeding; ③ combined with other severe primary diseases or severely impaired hepatic or renal function; ④ abnormal cervical smear; ⑤ endometrium thickness by vaginal ultrasound >5 mm; ⑥ a history of antidepressant drugs, antipsychotic drugs or drugs containing other components in the past 3 months.

### 2.4. Methods

#### 2.4.1. Medication and Administration

Patients in the treatment group were administered MEG (10 g preparation, 1 bag daily taken twice) for 12 consecutive weeks. The placebo group received placebo (starch, dextrin, bitterant, and so on, it is appearance is very close to that of MEG) as in the treatment group. All trial drugs, including treatment and placebo types, were provided by Huarun 39 medical Limited through Share Ltd. They were packaged according to the requirements of blinding and met quality requirements.

#### 2.4.2. Observation Indexes

*(1) Efficacy Indexes*. The primary efficacy measure in this trial was the VHS [15], which includes vaginal elasticity, moisture, pH, mucosa and discharge. Scores for each item ranged from 1 to 4 points. The lower the score, the severer the local vaginal symptom. Scores were recorded by a doctor while performing gynecologic examination.

Secondary efficacy parameters included vaginitis score, VMI, FSFI, and modified KI.

Vaginitis score [8] was based on the patient's subjective feelings, including vaginal pain, painful sexual intercourse, vaginal itching, and burning and ranged from 0 to 3 points. The higher the score, the more serious the symptom.

For assessing the VMI [8], the vagina was dilated with a vaginal speculum. Aseptic cotton bubs were soaked in physiological saline, extended into the upper 1/3 vaginal wall and gently rolled, removed, transversely put on the slide, and rolled in one direction. After fixation with 95% ethanol for 30 minutes, Papanicolaou staining was performed, and the percentages of superficial and basal cells in the vaginal epithelium were determined, respectively.

The FSFI [16] included 19 items, with six domains of desire, arousal, lubrication, orgasm, satisfaction, and pain, which mainly reflect feelings and reactions about sexual life in the last 4 weeks.

Based on the original Kupperman scale, two items were added to the modified KI [17], including urinary system symptoms and vulvovaginal discomfort. The modified KI included 13 entries reflecting menopause symptoms. There were different basic and severity scores in each entry. Severity score ranged from 0 to 3 points; symptom score=Basic score×Severity score. The total score was derived as the sum of all symptom scores.

*(2) Safety Parameters*. Safety assessments included routine blood test, urine routine test, liver function test, renal function test, serum E_2_ and FSH level assessment, electrocardiography, breast ultrasound and pelvic ultrasound. Meanwhile, adverse events were recorded.

#### 2.4.3. Follow-Up

Efficacy indexes were evaluated at baseline, and at 4, 8, and 12 weeks during drug administration, as well as 4 weeks after drug withdrawal. Safety parameters, including laboratory examination and ultrasound, were obtained before group assignment and at 12 weeks during drug administration. Adverse events were observed during the whole follow-up period.

### 2.5. Statistical Analysis

The SPSS 23.0 software was used for statistical analysis. Normality and homogeneity of variance were assessed for VHS, vaginitis score, VMI, and modified KI. Normally distributed data were assessed by independent two-sample t-test, with paired sample t-test used for within-group comparison before and after treatment. For nonnormally distributed parameters, the nonparametric test was used for comparing groups before and after treatment.

## 3. Results

### 3.1. Baseline Patient Characteristics

A total of 88 patients were enrolled in this study, including 1 case of mistaken identity, 1 without medication record, 11 lost to follow up and 75 cases who completed treatment (39 and 36 cases in the treatment and placebo groups, respectively). All the enrolled patients met the set inclusion criteria. There was no statistical differences in baseline indexes, including demographic data (age, body mass index, menopause time and so on) and efficacy indexes (VHS, vaginitis score, VMI, FSFI, and modified KI). The baseline parameters of the two groups were comparable (Table 1).

### 3.2. Efficacy

#### 3.2.1. Vaginal Health Score

All patients were followed up during the medication period and at 4 weeks after drug withdrawal. VHS in the treatment group were significantly increased in comparison with baseline values (*p*<0.05). VHS in the placebo group after treatment and during the follow-up period were not statistically different from baseline values (*p*>0.05). During the medication period and at 4 weeks after drug withdrawal, VHS in the treatment group were markedly increased in comparison with those of the placebo group (*p*<0.05).

During the medication period and at 4 weeks after drug withdrawal, vaginal moisture, pH, mucous, and discharge scores in the treatment group were significantly increased in comparison with baseline values (*p*<0.05). After 4 weeks of treatment, the vaginal elasticity score in the treatment group was increased in comparison with the baseline value, but the difference was not statistically significant (*p*>0.05). After 8 and 12 weeks of treatment as well as at 4 weeks after drug withdrawal, vaginal elasticity scores in the treatment group were significantly increased in comparison with baseline values (*p*<0.05). During the medication period and follow-up, vaginal elasticity, moisture, pH, mucous, and discharge scores in the placebo group were not statistically different with those at baseline (*p*>0.05).

During the medication period and at 4 weeks after drug withdrawal, vaginal moisture, mucous, and discharge scores in the treatment group were significantly increased in comparison with those of the placebo group (*p*<0.05). After 4 and 8 weeks of treatment, vaginal elasticity and vaginal pH in the treatment group were increased in comparison with those of the placebo group, but the differences were not statistically significant (*p*>0.05). After 12 weeks of treatment and at 4 weeks after drug withdrawal, vaginal elasticity and pH in the treatment group were significantly higher than those of the placebo group (*p*<0.05) (Table 2)

#### 3.2.2. Vaginitis Score

During the medication period and 4 weeks after drug withdrawal, vaginitis scores in the treatment group were significantly decreased in comparison with baseline values (*p*<0.05). Vaginitis scores in the placebo group after medication and during the follow-up period were not statistically different from baseline values (*p*>0.05). Meanwhile, vaginitis scores in the treatment group during the medication period and 4 weeks after drug withdrawal were starkly decreased in comparison with those of the placebo group (*p*<0.05).

During the medication period and 4 weeks after drug withdrawal, vaginal pain, vaginal itching, and burning scores in the treatment group were significantly decreased in comparison with baseline values (all* p*<0.05). After 4 and 8 weeks of treatment, painful sexual intercourse scores in the treatment group were decreased in comparison with baseline values, but the differences were not statistically significant (*p*>0.05); after 12 weeks of treatment and 4 weeks after drug withdrawal, painful sexual intercourse scores in the treatment group were significantly decreased in comparison with baseline values (*p*<0.05). Vaginal pain, painful sexual intercourse, vaginal itching, and burning scores in the placebo group in the medication period and during follow-up were not statistically different from those at baseline (*p*>0.05). In the medication period and 4 weeks after medicine withdrawal, vaginal pain and burning scores in the treatment group were markedly decreased in comparison with those of the placebo group (*p*>0.05). After 4 weeks of treatment, vaginal itching scores in the treatment group were decreased in comparison with those of the placebo group, but the difference was not statistically significant (*p*>0.05). After 8 and 12 weeks of treatment as well as 4 weeks after drug withdrawal, vaginal itching scores in the treatment group were significantly lower than those of the placebo group (*p*<0.05). After 4 and 8 weeks of treatment, respectively, painful sexual intercourse scores in the treatment group were decreased in comparison with those of the placebo group, but the differences were not statistically significant (*p*>0.05). After 12 weeks of treatment, painful sexual intercourse scores in the treatment group were significantly lower than those of the placebo group (*p*<0.05). At 4 weeks after drug withdrawal, there were no statistical differences in painful sexual intercourse scores between the treatment and placebo groups (*p*>0.05) (Table 3)

#### 3.2.3. Vaginal Maturation Index

During the medication period and 4 weeks after drug withdrawal, the percentage of superficial cells in the vaginal epithelium was markedly increased while that of basal cells was significantly reduced in the treatment group in comparison with values before treatment (*p*<0.05). In the medication period and during follow-up, the percentages of superficial and basal cells in the vaginal epithelium were not statistically different from baseline values in the placebo group (*p*>0.05). However, 4 weeks after drug withdrawal, the percentages of basal cells in the vaginal epithelium in the placebo group were significantly increased in comparison with the baseline value (*p*<0.05). In the medication period and 4 weeks after drug withdrawal, the percentages of superficial cells in the vaginal epithelium in the treatment group were starkly increased while those of basal cells were significantly decreased, in comparison with those of the placebo group (*p*<0.05) (Table 4).

#### 3.2.4. Female Sexual Function Index

During the medication period and 4 weeks after drug withdrawal, the FSFI in the treatment group was significantly increased in comparison with the baseline value (*p*<0.05). FSFI in the placebo group in the medication period and during follow-up were not statistically different from those at baseline (*p*>0.05). In the medication period and 4 weeks after drug withdrawal, there was no significant difference in FSFI between the treatment and placebo groups (*p*<0.05).

During the medication period and 4 weeks after drug withdrawal, lubrication scores in the treatment group were significantly increased in comparison with baseline values (*p*<0.05). Satisfaction scores in the treatment group were significantly increased in the medication period compared with baseline values (*p*<0.05) but not at 4 weeks after drug withdrawal (*p*>0.05). After 12 weeks of treatment and 4 weeks after drug withdrawal, painful sexual intercourse scores in the treatment group were starkly increased in comparison with baseline values (*p*<0.05). In the medication and follow-up periods, desire, subjective arousal ability and orgasm scores in the treatment group were not statistically different from baseline values (*p*>0.05). The six domains of the FSFI in the placebo group in the medication and follow-up periods were not statistically different from baseline values (*p*>0.05). In the medication period and during follow-up,no significant differences in the 6 domains of the FSFI between the treatment and placebo groups (*p*>0.05) (Table 5).

#### 3.2.5. Modified Kupperman Index

In the medication period and 4 weeks after drug withdrawal, modified KI in the treatment group were significantly decreased in comparison with baseline values (*p*<0.05). Modified KI in the placebo group in the medication and follow-up periods were not statistically significant from baseline values (*p*>0.05). After 4 weeks of treatment, modified KI in the treatment group were decreased in comparison with those of the placebo group, but the difference was not statistically significant (*p*>0.05). After 8 and 12 weeks of treatment, as well as 4 weeks after drug withdrawal, modified KI in the treatment group were significantly decreased in comparison with those of the placebo group (*p*< 0.05).

In the medication period and 4 weeks after drug withdrawal, scores for hot flashes, sweating, insomnia, fatigue and urinary tract infection in the treatment group were markedly decreased in comparison with baseline values (*p*<0.05). After 4 weeks of treatment, arthralgia and myalgia scores in the treatment group were decreased in comparison with baseline values, but the difference was not statistically significant (*p*>0.05). After 8 and 12 weeks of treatment, as well as 4 weeks after drug withdrawal, these scores were significantly decreased in the treatment group in comparison with baseline values (*p*<0.05). In the medication period, paresthesia, nervousness, melancholia, vertigo, headache, heart palpitation, sexual complaints scores in the treatment group were not statistically different from those at baseline (*p*>0.05). In the medication and follow-up period, modified KI in the placebo group were not statistically different from baseline values (*p*>0.05). In the medication period, scores for hot flashes, sweating, insomnia and fatigue in the treatment group were significantly decreased in comparison with those of the placebo group (*p*<0.05). At 4 weeks after drug withdrawal, fatigue scores in the treatment group remained significantly lower than those of the placebo group (*p*<0.05); however, no significant differences in scores for hot flashes, sweating and insomnia were found between the treatment and placebo groups (*p*>0.05). After 4 and 8 weeks of treatment, paresthesia scores in the treatment group were decreased in comparison with those of the placebo group, but the differences were not statistically significant (*p*>0.05). After 12 weeks of treatment and 4 weeks after drug withdrawal, paresthesia scores in the treatment group were significantly lower than those of the placebo group (*p*<0.05). After 4 weeks of treatment, bone and joint pain scores in the treatment group were decreased in comparison with those of the placebo group, but the difference was not statistically significant (*p*>0.05). After 8 and 12 weeks of treatment as well as 4 weeks after drug withdrawal, Arthralgia and myalgia scores in the treatment group were significantly lower than those of the placebo group (*p*<0.05). In the medication and follow-up periods, there was no statistical difference in modified KI between the treatment and placebo groups (Table 6).

### 3.3. Safety Parameters

#### 3.3.1. Changes in Serum Hormone Levels, Endometrial Thickness, and Breast Ultrasound

At 12 weeks of treatment, serum E_2_, and FSH levels, endometrial thickness and breast ultrasound findings for both groups were within the normal ranges after menopause.

#### 3.3.2. Laboratory Indexes

At 12 weeks of treatment, no significant differences were found in blood routine, urine routine, hepatic and renal function parameters, as well as electrocardiograms between the two groups.

#### 3.3.3. Adverse Events

During the medication period, adverse events occurred in 10 cases and were all mild or moderate. Of these, 2 cases were associated with medication, including 1 each in the treatment (diarrhea) and placebo (abdominal distention) groups, respectively; both cases were mild, and symptoms were relieved by taking medicine after a meal. No vaginal bleeding was found in this study. There was no case of withdrawal due to adverse drug reactions (Table 7).

## 4. Discussion

VVA is a common and frequently encountered disease in menopausal women. Unlike the symptoms of hot flashes, sweating, depression and anxiety, VVA is not relieved with time, and seriously affects the patients' quality of life.

From the Chinese medicine perspective, menopausal symptoms are thought to be associated with a decline in kidney Yin or Yang or both. Kidney is the congenital life basis and closely related to reproduction physiological activities. TCM believes that the menstruation, pregnancy, delivery, and lactation in woman's life are all supported by kidney Yin. Excessive consumption of kidney Yin such as irregular periods, repeated pregnancy, and long time lactation will lead to kidney Yin deficiency. It is typically characterized by syndrome such as vaginal dryness, hot flushes and night sweats, dizziness, and insomnia. According to TCM theory, Yin and Yang grow together and nourish each other. It will be accompanied by kidney Yang deficiency as kidney Yin is further exhausted with stages of menopause. Women may present with symptoms such as vaginal burning and itching, aversion to cold, urine incontinence, and loose motions.

MEG are composed of* Eclipta*,* Fructus Ligustri Lucidi*, Cynomorium Songaricum,* Herba Cistanches,* and* Cortex Phellodendri*. The efficacy of* Fructus Ligustri Lucidi* and* Eclipta* is nourishing kidney Yin, so as to moisten the vagina.* Herba Cistanches* and* Cynomorium Songaricum* are often used for warming kidney Yang.* Cortex Phellodendri* has the function of clearing heat, relieving toxicity and diminishing inflammations, and it has antipruritic properties in patients with vulvovaginal atrophy. Therefore, the combination of these herbs is very effective for vulvovaginal atrophy due to deficiency of kidney Yin and Yang which is accompanied with systemic symptoms.

The current study showed that MEG obviously improved the symptoms of reduced vaginal discharge, pale mucous membrane and reduced vaginal elasticity, relieved vaginal pain, painful sexual intercourse, and vaginal itching and burning, while increasing the percentage of epithelial cells and decreasing that of basal cells in the vaginal epithelium. At 12 weeks of medication with MEG, vaginal dryness and itching scores were decreased by 1.18 and 1.01 points, respectively; the percentages of superficial and basal cells in the vaginal epithelium increased by 4.82% and decreased by 21.38%, respectively. Previous studies showed that 12 weeks after treatment with vaginal E_2_ soft capsule (10 *μ*g), vaginal dryness and itching scores decrease by 1.5 and 0.8 points, respectively; meanwhile, the percentages of superficial and basal cells in the vaginal epithelium increased by 17% and decreased by 44%, respectively [18]. After 12 weeks of Ospemifene administration (60 mg), the vaginal dryness score decreased by 1.3 points; the percentages of superficial and basal cells in the vaginal epithelium increased by 7% and decreased by 31.7%, respectively [19]; at 12 weeks of dehydroepiandrosterone sulfate use (DHEA, 6.5 mg), the vaginal dryness score decreased by 1.44 points; the percentages of superficial and basal cells in the vaginal epithelium increased by 8.44% and decreased by 27.7%, respectively [20]. The above results indicated that the effects of MEG on vaginal dryness and vaginal itching were significant, and equivalent to those of local vaginal estrogens, Ospemifene, and vaginal DHEA, although VMI improvement was weaker compared with what found for the above drugs. In addition, MEG also improved female sexual function, mainly reflected in increased vaginal moisture, enhanced sexual life satisfaction, and relieved painful sexual intercourse, with significant differences compared with baseline values; however, there were no significant differences in comparison with the values of the placebo group, which might be related to the small sample size of this study. Therefore, larger sample studies are needed to draw a definite conclusion.

Cases with VVA are often accompanied by other menopausal symptoms. A study showed that vaginal dryness is significantly related to the occurrence of hot flashes (OR=1.52; 95%CI, 1.19-1.93) [21]. The duration of vasomotion is often over 5.5 years [22], and more than 50% of patients still have symptoms 4 years after menopause [21], which increases the risk of combined vulvovaginal symptoms. Karmakar N [23] found incidence rates of hot flashes, sweating, insomnia and fatigue among menopausal women in West Bengal (India) of 60%, 84%, and 93%, respectively. At 6 months after Ospemifene use (60 mg), changes in systemic symptoms such as insomnia, anorexia, dyspnea, nausea, and vomiting were shown not to differ from those of the placebo group [24]; the symptom of hot flashes was aggravated in 10% of patients after long-term Ospemifene administration, with 2% of subjects discontinuing the drug because of nontolerance [25]. In the present study, MEG significantly reduced the systemic symptoms of menopause syndrome, including vasomotor, and somatic symptoms and improved hot flashes, sweat, insomnia, fatigue, and bone and joint pain; the effects were obviously better than those of placebo. Li [26] found that Echinacetin (ECH), an extract from Herba Cistanche, has antiaging effect by increasing the antistress ability, increasing the levels of IL-2 in serum and NO and SOD in brain tissue, reducing the levels of IL-6 and MDA in brain tissue. Guo [27] found that intragastric administrate* Cynomorium Songaricum *in rats can alleviate the effect of overexercise on serum testosterone and corticosterone, promote the synthesis of protein, inhibit breakdown of amino acid and protein, and increase hemoglobin content and glycogen stores. These effects may help improve insomnia, fatigue, and joint pain in postmenopausal women. Moreover, MEG did not affect serum E_2_ and FSH levels, and no damage to hepatic and renal function or serious adverse reactions occurred.

In the present study, recurrence in patients was followed up 4 weeks after drug withdrawal, and local manifestations in the vagina (vaginal elasticity, vaginal moisture, vaginal mucous and so on), atrophy symptoms (vaginal pain, itching, burning, and so on) and systemic symptoms (fatigue, bone and joint pain and so on) remained different from those of the placebo group. However, there are no reliable data about recurrence after withdrawal of other drugs such as Ospemifene, DHEA, and local estrogens.

Modern pharmacology found that the constituents of MEG, such as* Fructus Ligustri Lucidi*,* Eclipta*,* Herba Cistanches,* and* Cynomorium Songaricum*, have obvious estrogenic effects. There are terpenes, flavonoids, and coumaric esters in* Fructus Ligustri Lucidi* and* Eclipta*, with structures similar to that of estradiol [28–31]; they could induce the expression of the ERE-luciferase reporter gene and act as estrogens. Zheng [32] found that, compared with the blank control group, use of Er Zhi Wan (1.365 g/kg) results in overt keratinization of epithelial cells in the vaginal epithelium, epithelial thickening, increased ER*α*, and ER*β* amounts in vaginal tissues, and selectively upregulated ER*β* in uterine tissues; moreover, Er Zhi Wan does not significantly affect ER*α* levels and serum estrogen content in ovariectomized rats. Wang [33] found that the main active ingredients echinacoside and acteoside could bind the estrogen receptors ER*α* and ER*β*, increase the luciferase activity of ERE and play estrogenic roles. In addition, according to the upregulation of both components in the luciferase activity of ERE, selectivity of acteoside for ER*α* and ER*β* is not significant, and echinacoside has higher affinity to ER*β*. The regulatory effects of these selective estrogen receptors might be the molecular mechanism by which MEG effectively improved the symptoms of VVA, increasing VMI, and relieve the symptoms of hot flashes and sweating without endometrial thickening and changing serum hormone levels [34].

Here, a randomized, double-blind, placebo-controlled clinical trial was firstly conducted for the traditional Chinese medicine treating VVA. VHS, vaginitis score, and VMI are all commonly used parameters for VVA evaluation worldwide. Vaginal atrophy was comprehensively evaluated from the objective and subjective aspects; the FSFI has been the most frequently used female function scale in the past few decades, with good evaluation validity, credibility, and reliability [16]. The modified KI is of high value for the evaluation of systemic symptoms in the menopause period [17]. By adopting the above scales, the clinical efficacy of the MEG was assessed comprehensively and systematically.

There were limitations in this study. First, only related symptoms were assessed in patients 4 weeks after drug withdrawal, and the duration of curative effect was not determined. Therefore, the follow-up time should be increased to further characterize MEG. Secondly, the sample size was relatively small, and subgroup analysis was not performed on age and symptom severity; in addition, efficacy indexes, such as sexual function dimensions in some females, were not consistent with those of the placebo group and before medication. Therefore, sample size should be further expanded. Finally, this study did not include a positive drug group. Therefore, similar trials are underway in our group to further compare the clinical efficacy of MEG with that of local estrogens in the treatment of VVA.

## 5. Conclusion

(1) Use of MEG for 4 weeks could obviously improve vaginal elasticity and vaginal moisture, increase vaginal discharge, decrease vaginal pH, obviously relieve vaginal pain, itching, and burning, and improve vaginal health in patients with menopause-related VVA. Moreover, the curative effects persisted 4 weeks after drug withdrawal.

(2) Use of MEG for 8 weeks could obviously relieve painful sexual intercourse, and the curative effects were maintained after drug withdrawal; however, there was no obvious effect on sexual desire and subjective sexual arousal after administration of MEG.

(3) With the prolongation of medication time, MEG could significantly increase the percentage of superficial cells in the vaginal epithelium, while decreasing that of basal cells, without significantly altering serum estrogen levels.

(4) MEG could obviously improve menopause-related systemic symptoms such as hot flashes, sweating, insomnia, fatigue, and bone and joint pain.

(5) No adverse effects on the endometrium, breast, liver, and kidney were observed with continuous use of MEG for 12 weeks.

## Figures and Tables

**Table 1:** Baseline parameters in the two groups.

	treatment group	placebo group	*p*
demographic			
n	39	36	
Age, years	59.67±3.47	58.53±4.53	0.338
Body mass index, kg/m	24.23±3.45	24.59±3.51	0.726
age of menopause, years	49.43±3.93	49.44±4.25	0.061
Menopause time, years	10.24±5.37	9.09±5.96	0.241
Course of disease, years	1.56±0.88	1.21±1.21	0.979

vaginal health score	9.87±2.33	10.44±2.35	0.245
elasticity	2.18±0.56	2.25±0.55	0.585
moisture	2.00±0.76	2.08±0.73	0.700
pH	1.92±0.66	1.89±0.71	0.810
mucosa	1.95±0.89	2.14±0.72	0.330
discharge	1.82±0.68	2.08±0.73	0.127

Vaginitis score	4.50±1.57	4.58±1.57	0.925
pain	0.87±0.52	0.94±0.63	0.619
painful sexual intercourse	1.18±0.73	1.26±0.81	0.673
itching	1.38±0.75	1.19±0.58	0.276
burning	1.18±0.68	1.06±0.71	0.472

vaginal maturation index			
percentage of superficial cells	0.67±1.18	1.06±1.24	0.079
percentage of basal cells	55.23±10.32	53.53±9.95	0.494

female sexual function index	12.78±8.55	12.26±8.39	0.738
desire	2.38±0.76	2.40±0.98	0.841
arousal	1.65±1.66	1.48±1.55	0.710
lubrication	1.92±1.94	1.80±1.90	0.844
orgasm	1.81±1.82	1.58±1.62	0.527
satisfaction	2.79±0.89	3.01±1.01	0.212
pain	2.24±2.15	2.00±1.97	0.431

modified Kupperman Index	22.64±6.38	23.61±6.56	0.531
Sweating, hot flushes	1.08±0.81	1.14±0.80	0.796
Paresthesia	0.85±0.71	1.19±0.82	0.060
Insomnia	1.23±0.78	1.31±0.79	0.849
Nervousness	1.46±0.64	1.44±0.84	0.817
Melancholia	1.00±0.76	0.86±0.83	0.390
Vertigo	0.82±0.76	0.89±0.62	0.589
Fatigue	1.62±0.49	1.58±0.73	0.629
Arthralgia, myalgia	0.79±0.83	0.86±0.76	0.654
Headache	0.90±0.88	1.03±0.81	0.529
Heart palpitation	0.72±0.76	0.67±0.59	0.971
Formication	0.28±0.46	0.28±0.45	0.967
Sexual complaints	1.82±0.64	1.94±0.71	0.257
Urinary tract infection	0.74±0.85	0.56±0.65	0.410

**Table 2:** Vaginal health score.

	groups	baseline	4 weeks	8 weeks	12 weeks	4 weeks after drug withdrawal	*p*_1_	*p*_2_	*p*_3_	*p*_4_
VHS	treatment group	9.87±2.33	12.00±1.81	13.10±1.79	14.64±1.37	13.41±1.37	<0.001	<0.001	<0.001	<0.001
	placebo group	10.44±2.35	10.44±2.32	10.36±2.31	10.39±2.13	10.42±2.29	1.000	0.370	0.640	0.560
	*p*	0.245	0.002	<0.001	<0.001	<0.001				

elasticity	treatment group	2.18±0.56	2.21±0.57	2.31±0.57	2.82±0.39	2.77±0.43	0.32	0.03	<0.001	<0.001
	placebo group	2.25±0.55	2.25±0.55	2.22±0.54	2.22±0.54	2.22±0.54	1.000	0.320	0.320	0.320
	*p*	0.585	0.744	0.484	<0.001	<0.001				

moisture	treatment group	2.00±0.76	2.59±0.55	2.90±0.60	3.08±0.42	2.85±0.54	<0.001	<0.001	<0.001	<0.001
	placebo group	2.08±0.73	2.08±0.73	2.08±0.73	2.11±0.71	2.11±0.75	1.000	1.000	0.320	0.320
	*p*	0.700	0.001	<0.001	<0.001	<0.001				

pH	treatment group	1.92±0.66	2.10±0.60	2.23±0.67	2.59±0.59	2.54±0.60	<0.001	<0.001	<0.001	<0.001
	placebo group	1.89±0.71	1.94±0.71	1.94±0.67	1.92±0.69	1.92±0.73	0.160	0.160	0.320	0.320
	*p*	0.810	0.305	0.081	<0.001	<0.001				

mucosa	treatment group	1.95±0.89	2.54±0.60	2.77±0.48	2.97±0.28	2.85±0.43	<0.001	<0.001	<0.001	<0.001
	placebo group	2.14±0.72	2.08±0.69	2.03±0.61	2.08±0.55	2.06±0.58	0.160	0.160	0.480	0.260
	*p*	0.330	0.004	<0.001	<0.001	<0.001				

discharge	treatment group	1.82±0.68	2.56±0.55	2.90±0.45	3.18±0.39	2.41±0.50	<0.001	<0.001	<0.001	<0.001
	placebo group	2.08±0.73	2.08±0.73	2.08±0.73	2.06±0.67	2.11±0.75	1.000	1.000	0.560	0.320
	*p*	0.127	0.002	<0.001	<0.001	0.048				

*P*: significant difference between the treatment and placebo groups.

*P*_*1*_: significant difference in score and baseline between the treatment and placebo groups after 4 weeks of medication.

*P*_*2*_: significant difference in score and baseline between the treatment and placebo groups after 8 weeks of medication.

*P*_*3*_: significant difference in score and baseline between the treatment and placebo groups after 12 weeks of medication.

*P*_*4*_: significant difference in score and baseline between the treatment and placebo groups 4 weeks after drug withdrawal.

**Table 3:** Vaginitis scores.

	groups	baseline	4 weeks	8 weeks	12 weeks	4 weeks after drug withdrawal	*p*_1_	*p*_2_	*p*_3_	*p*_4_
Vaginitis score	treatment group	4.50±1.57	2.68±0.99	1.91±0.97	1.41±0.85	2.09±1.02	<0.001	<0.001	<0.001	<0.001
	placebo group	4.58±1.57	4.63±1.54	4.58±1.61	4.63±1.54	4.58±1.64	0.710	1.000	0.650	1.000
	*p*	0.925	<0.001	<0.001	<0.001	<0.001				

pain	treatment group	0.87±0.52	0.64±0.49	0.51±0.51	0.26±0.44	0.36±0.49	0.010	<0.001	<0.001	<0.001
	placebo group	0.94±0.63	0.97±0.65	0.97±0.65	0.94±0.63	1.00±0.68	0.320	0.320	1.000	0.480
	*p*	0.619	0.024	0.002	<0.001	<0.001				

painful sexual intercourse	treatment group	1.18±0.73	1.09±0.68	1.00±0.62	0.95±0.58	0.95±0.58	0.160	0.050	0.030	0.030
	placebo group	1.26±0.81	1.32±0.82	1.37±0.83	1.37±0.83	1.32±0.82	0.320	0.160	0.160	0.320
	*p*	0.673	0.272	0.076	0.046	0.080				

itching	treatment group	1.38±0.75	0.85±0.63	0.38±0.49	0.23±0.43	0.49±0.56	<0.001	<0.001	<0.001	<0.001
	placebo group	1.19±0.58	1.08±0.55	1.08±0.65	1.06±0.63	1.08±0.60	0.050	0.250	0.100	0.210
	*p*	0.276	0.086	<0.001	<0.001	<0.001				

burning	treatment group	1.18±0.68	0.28±0.51	0.08±0.27	0.03±0.16	0.44±0.50	<0.001	<0.001	<0.001	<0.001
	placebo group	1.06±0.71	1.08±0.69	1.11±0.71	1.08±0.73	1.03±0.81	0.560	0.320	0.560	0.650
	*p*	0.472	<0.001	<0.001	<0.001	<0.001				

**Table 4:** Vaginal maturation index.

	groups	baseline	4 weeks	8 weeks	12 weeks	4 weeks after drug withdrawal	*p*_1_	*p*_2_	*p*_3_	*p*_4_
percentage of superficial cells	treatment group	0.67±1.18	2.39±0.79	4.28±1.88	5.49±1.64	4.59±1.68	<0.001	<0.001	<0.001	<0.001
	placebo group	1.06±1.24	2.42±0.96	0.86±0.96	0.86±0.96	0.86±1.25	0.410	0.050	0.110	0.040
	*p*	0.079	<0.001	<0.001	<0.001	<0.001				

percentage of basal cells	treatment group	55.23±10.32	44.56±10.74	37.31±8.54	33.85±7.91	41.26±8.48	<0.001	<0.001	<0.001	<0.001
	placebo group	53.53±9.95	53.22±9.79	54.58±9.14	55.17±9.09	57.53±8.78	0.880	0.450	0.180	0.010
	*p*	0.494	0.001	<0.001	<0.001	<0.001				

**Table 5:** Female sexual function index.

	groups	baseline	4 weeks	8 weeks	12 weeks	4 weeks after drug withdrawal	*p*_1_	*p*_2_	*p*_3_	*p*_4_
FSFI	treatment group	12.79±8.55	13.09±8.83	13.45±9.05	13.76±9.36	13.46±9.16	<0.001	<0.001	<0.001	<0.001
	placebo group	12.26±8.39	12.47±8.33	12.42±8.48	12.38±8.52	12.27±8.43	0.549	0.905	0.411	0.190
	*p*	0.738	0.628	0.352	0.236	0.325				

desire	treatment group	2.39±0.76	2.77±1.90	2.40±0.76	2.43±0.78	2.42±0.79	0.527	0.317	0.059	0.102
	placebo group	2.40±0.98	1.11±1.35	2.42±0.97	2.43±0.99	2.42±0.98	0.317	0.564	1.000	0.564
	*p*	0.841	0.778	0.930	0.947	0.960				

arousal	treatment group	1.65±1.66	1.62±1.65	1.60±1.64	1.61±1.64	1.58±1.63	0.527	0.705	1.000	0.317
	placebo group	1.48±1.55	1.53±1.56	1.53±1.56	1.54±1.57	1.52±1.55	0.705	0.705	0.293	1.000
	*p*	0.710	0.818	0.924	0.951	0.906				

lubrication	treatment group	1.92±1.94	2.13±2.16	2.45±2.42	2.47±2.45	2.41±2.39	<0.001	<0.001	<0.001	<0.001
	placebo group	1.80±1.90	1.89±1.94	1.89±1.94	1.89±1.94	1.89±1.92	0.102	0.102	0.102	0.194
	*p*	0.844	0.587	0.246	0.226	0.260				

orgasm	treatment group	1.81±1.82	1.78±1.83	1.79±1.82	1.77±1.80	1.76±1.80	0.157	0.180	0.705	1.000
	placebo group	1.58±1.62	1.63±1.63	1.63±1.64	1.63±1.63	1.62±1.64	0.564	0.655	0.739	1.000
	*p*	0.527	0.644	0.597	0.669	0.698				

satisfaction	treatment group	2.79±0.89	2.92±0.87	2.93±0.88	3.02±0.94	2.89±0.86	0.028	0.044	0.006	0.144
	placebo group	3.01±1.01	3.05±0.96	3.01±1.03	2.98±1.01	2.94±1.03	0.380	0.739	0.448	0.234
	*p*	0.212	0.404	0.749	0.888	0.741				

pain	treatment group	2.24±2.15	2.25±2.23	2.29±2.26	2.47±2.42	2.40±2.36	0.102	0.067	0.005	0.020
	placebo group	2.00±1.97	1.95±1.98	1.95±2.00	1.90±1.95	1.89±1.92	0.317	0.414	0.323	0.283
	*p*	0.431	0.349	0.297	0.225	0.255				

**Table 6:** Modified Kupperman Index.

	groups	baseline	4 weeks	8 weeks	12 weeks	4 weeks after drug withdrawal	*p*_1_	*p*_2_	*p*_3_	*p*_4_
modified KI	treatment group	22.64±6.38	19.23±6.16	17.72±6.17	17.38±5.67	19.18±5.84	<0.001	<0.001	<0.001	<0.001
	placebo group	23.61±6.56	23.47±7.17	22.75±6.18	23.19±6.05	23.25±5.66	0.219	0.136	0.571	0.978
	*p*	0.531	0.054	0.002	<0.001	0.007				

sweating, hot flushes	treatment group	4.31±3.23	2.77±2.92	2.26±3.02	2.15±3.02	2.87±2.89	<0.001	<0.001	<0.001	0.001
	placebo group	4.56±3.19	4.56±3.19	4.44±3.28	4.44±3.28	4.56±3.19	1.000	0.317	0.317	1.000
	*p*	0.796	0.036	0.007	0.004	0.053				

paresthesia	treatment group	1.69±1.42	1.59±1.39	1.59±1.39	1.38±1.39	1.59±1.39	0.317	0.317	0.058	0.480
	placebo group	2.39±1.64	2.17±1.61	2.11±1.72	2.39±1.71	2.33±1.69	0.102	0.059	0.705	0.527
	*p*	0.060	0.058	0.110	0.004	0.026				

insomnia	treatment group	2.46±1.55	1.85±1.33	1.69±1.34	1.69±1.26	1.95±1.41	0.001	0.001	0.001	0.022
	placebo group	2.61±1.57	2.72±1.45	2.33±1.62	2.22±1.64	2.33±1.55	0.157	0.157	0.109	0.285
	*p*	0.849	0.012	0.042	0.047	0.126				

nervousness	treatment group	2.92±1.29	2.77±1.35	2.77±1.35	2.72±1.34	3.03±1.37	0.083	0.059	0.096	1.000
	placebo group	2.89±1.69	2.72±1.80	2.61±1.71	2.72±1.80	2.67±1.59	0.180	0.257	0.480	0.317
	*p*	0.817	0.409	0.542	0.554	0.322				

melancholia	treatment group	1.00±0.76	1.05±0.82	1.05±0.82	1.05±0.82	1.08±0.80	1.000	1.000	1.000	0.564
	placebo group	0.86±0.83	0.86±0.73	0.89±0.72	0.91±0.78	0.94±0.77	0.157	0.083	0.157	0.059
	*p*	0.390	0.347	0.440	0.539	0.551				

vertigo	treatment group	0.82±0.76	0.83±0.81	0.75±0.74	0.85±0.83	0.88±0.72	1.000	0.317	0.527	0.257
	placebo group	0.89±0.62	0.91±0.61	0.89±0.63	0.94±0.59	1.06±0.59	1.000	0.655	0.705	0.059
	*p*	0.589	0.419	0.329	0.406	0.227				

fatigue	treatment group	1.62±0.49	1.18±0.55	1.05±0.55	1.00±0.60	1.08±0.62	<0.001	<0.001	<0.001	<0.001
	placebo group	1.58±0.73	1.57±0.74	1.6±0.74	1.6±0.74	1.57±0.74	1.000	0.564	0.564	1.000
	*p*	0.629	0.017	0.001	<0.001	0.005				

arthralgia, myalgia	treatment group	0.79±0.83	0.73±0.75	0.53±0.64	0.50±0.60	0.53±0.64	0.317	0.004	0.002	0.004
	placebo group	0.86±0.76	0.86±0.73	0.91±0.70	0.91±0.70	0.91±0.74	0.317	0.564	0.655	0.655
	*p*	0.654	0.413	0.014	0.009	0.020				

headache	treatment group	0.90±0.88	0.90±0.81	0.90±0.78	0.95±0.78	1.00±0.82	1.000	1.000	0.564	0.356
	placebo group	1.03±0.81	1.03±0.82	1.03±0.75	1.03±0.75	0.97±0.79	1.000	1.000	1.000	0.480
	*p*	0.529	0.567	0.500	0.710	0.817				

heart palpitation	treatment group	0.72±0.76	0.80±0.82	0.65±0.74	0.65±0.70	0.73±0.68	0.257	0.257	0.366	1.000
	placebo group	0.67±0.59	0.66±0.59	0.60±0.60	0.63±0.60	0.60±0.55	1.000	0.527	0.739	0.527
	*p*	0.971	0.654	0.962	0.943	0.503				

formication	treatment group	0.28±0.46	0.35±0.62	0.25±0.44	0.28±0.45	0.28±0.45	0.317	0.317	1.000	1.000
	placebo group	0.28±0.45	0.29±0.46	0.29±0.46	0.26±0.44	0.20±0.41	1.000	1.000	0.317	0.18
	*p*	0.967	0.840	0.729	0.862	0.451				

sexual complaints	treatment group	3.64±1.29	3.69±1.17	3.54±1.17	3.54±1.17	3.54±1.17	0.564	0.317	0.414	0.527
	placebo group	3.89±1.43	3.83±1.46	3.94±1.39	4.00±1.35	4.00±1.35	0.317	0.564	0.157	0.157
	*p*	0.257	0.709	0.174	0.109	0.109				

urinary tract infection	treatment group	1.49±1.70	0.97±1.58	0.72±1.41	0.67±1.40	0.67±1.40	0.004	<0.001	<0.001	<0.001
	placebo group	1.11±1.30	1.06±1.31	1.11±1.30	1.11±1.30	1.11±1.30	0.317	1.000	1.000	0.317
	*p*	0.410	0.802	0.185	0.122	0.088				

**Table 7:** Adverse events.

adverse event	treatment group			placebo group			total		
	mild	moderate	severe	mild	moderate	severe	mild	moderate	severe
cough	1	0	0	0	0	0	1	0	0
pneumonia	1	0	0	0	0	0	1	0	0
diarrhea	1	0	0	1	0	0	2	0	0
knee arthritis	0	0	0	0	1	0	0	1	0
abdominal distention	0	0	0	1	0	0	1	0	0
zoster	0	0	0	0	1	0	0	1	0
hypertension	0	1	0	0	0	0	0	1	0
cold	0	0	0	1	0	0	1	0	0
somnolence	0	0	0	1	0	0	1	0	0

## Data Availability

The data used to support the findings of this study are available from the corresponding author upon request.
